# Developing a model for health determinants research within local government: lessons from a large, urban local authority

**DOI:** 10.12688/wellcomeopenres.17195.2

**Published:** 2022-06-21

**Authors:** Jane West, John Wright, Sally Bridges, Chris Cartwright, Kayley Ciesla, Kate E. Pickett, Robert Shore, Phil Witcherley, Mathew Flinders, Rosemary R.C. McEachan, Mark Mon-Williams, Pippa Bird, Laura Lennon, Duncan Cooper, Sarah Muckle, Kersten England, Trevor Sheldon

**Affiliations:** 1 Bradford Institute for Health Research, Bradford Hospitals National Health Service Trust, Bradford, BD9 6RJ, UK; 2 ScHARR, University of Sheffield, Sheffield, S1 4DA, UK; 3 Health Sciences, University of York, UK, York, YO10 5DD, UK; 4 Bradford Metropolitan District Council, Bradford, BD1 1HX, UK; 5 Dept of Politics & International Relations, University of Sheffield, Sheffield, S10 2TU, UK; 6 School of Psychology, University of Leeds, Leeds, LS2 9JT, UK; 7 Institute of Population Health Sciences, Queen Mary University of London, London, E1 2AB, UK

**Keywords:** Local Government, research system, public policy, evidenced based policy

## Abstract

**Background:** Socio-economic, cultural and environmental conditions strongly affect health across the life course. Local government plays a key role in influencing these wider determinants of health and levels of inequality within their communities. However, they lack the research infrastructure and culture that would enable them to develop an evidence-based approach to tackling the complex drivers of those conditions.

**Methods:** We undertook a scoping project using some research methods and some descriptive summaries to explore the potential for, and what would be needed to develop a local authority research system for the City of Bradford, UK. This included identifying the current research landscape and any barriers and enablers to research activity within the local authority using qualitative individual and focus group interviews, a rapid review of existing local research system models, scoping and description of the use of evidence in decision making and training opportunities and existing support for local government research.

**Results:** We identified four key themes important to developing and sustaining a research system: leadership, resource and capacity, culture, partnerships. Some use of research in decision making was evident but research training opportunities within the local authority were limited. Health research funders are slowly adapting to the local government environment, but this remains limited and more work is needed to shift the centre of gravity towards public health, local government and the community more generally.

**Conclusions:** We propose a model for a local authority research system that can guide the development of an exemplar whole system research framework that includes research infrastructure, data sharing, research training and skills, and co-production with local partners, to choose, use, generate, and deliver research in local government.

## Introduction

Socio-economic, cultural and environmental conditions strongly affect health across the life course and drive inequalities ^1, 2^. Addressing these wider conditions can improve health outcomes ^3^ and generate economic benefits ^4^ and local government plays a key role in influencing these conditions. Whilst the National Health Service (NHS) benefits from well-developed research infrastructure and culture, with strong university links, most of this has a clinical and biomedical focus and only 5% of research spending supports researching how to prevent poor health ^5^. Many of the wider determinants of health and potential for prevention research fall within the remit of local government, which lacks the formal research resources, structures, evidence culture and connection with National Institute for Health Research (NIHR) infrastructure. Developing these in local authorities, could facilitate choosing and using evidence to inform decisions, generating new knowledge, and evaluating attempts to improve outcomes. Being better users and producers of evidence could then result in better use of resources and savings, a priority when budgets are so tight. However, this is challenging, as local authorities work across whole systems that interact in complex ways. They are subject to changes in political leadership and direction, and quick wins may take priority over longer term public health impact. Local government-based knowledge generation is methodologically, logistically and politically complicated, requiring approaches which provide timely results for a real-world context often with a focus on improving rather than proving ^6^, and on systems rather than on areas or target groups.

Bradford is a post-industrial city in the North of England with high levels of deprivation and poor health, and a multi-ethnic population including a large Pakistani community and growing communities of East European and Roma people. Almost a quarter of children are growing up in poverty and the city has the 6 ^th^ lowest employment rate in England ^7^. Bradford is governed locally by Bradford Metropolitan District Council (BMDC), the 5 ^th^ largest metropolitan council in England.

Over the last 15 years, health and social researchers at Bradford Institute for Health Research (BIHR) have laid the foundations for public health research in close partnership with BMDC and collaborating universities. BMDC’s involvement in research, though significant, has mainly been responsive – supporting positively when approached, rather than using and creating research independently. For BMDC to fulfil its potential as a research user and generator, a research system that can deliver a shift change in culture, infrastructure, funding and activity is needed. Some of this potential was highlighted during the coronavirus disease 2019 (COVID-19) pandemic where local authorities have taken a leading role and increasingly want high quality linked data, to ask research questions, and to use and share research findings to plan and inform recovery. This means that they may now be more receptive to the concept of a formal local research system at the heart of decision making than ever before. Bradford’s engaged local authority, strong NIHR infrastructure and city-wide data linkage offers a research system testbed to develop local capacity as well as generalisable guidance for others at an earlier or similar stage in their research journey.

### Aims and objectives

In this scoping project, we set out to review current research activity within BMDC and explore a potential framework for a local research system, including what would be needed to put a system in place and how best to sustain it.

We had three specific objectives: 1) to better understand the current research landscape and any barriers and enablers to research activity within BMDC; 2) to review existing research system models for local government and use these to select or propose a system model; and 3) to explore how sustainable a research system might be through political cycles and budgetary challenges, and how to bring together local government, academic centres, NHS organisations and voluntary, cultural and commercial sectors within a local research system.

## Methods

**Figure 1.**
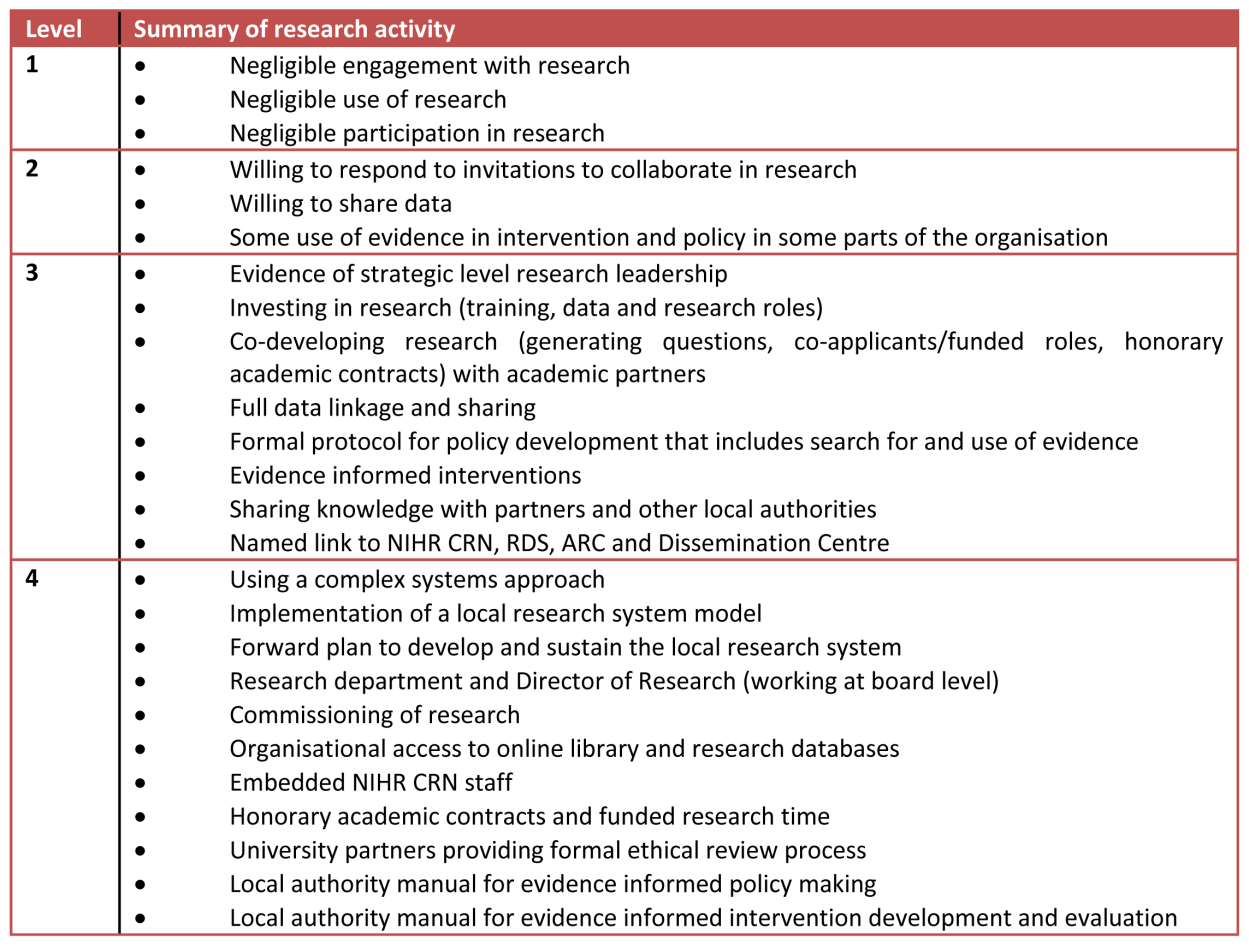
Typology of local authority research activity. NIHR CRN, National Institute for Health Research Clinical Research Network; RDS, Research Design Service; ARC, Applied Research Collaboration; LA, local authority.

In this project we used a range of both research and scoping approaches. Research components included an online survey and qualitative interviews and scoping activities included descriptive summaries of current activity and processes. We were interested to understand the perspectives of BMDC staff and leaders on the use of research, and the challenges and barriers to further developing this. We undertook an online survey of BMDC staff (n= 197 almost 40% response rate) to get a picture of existing research activity at a crude level across the organisation. We undertook qualitative focus group interviews (mixed levels/departments staff), and individual interviews with key BMDC staff (including the Chief Executive and Council Leader to gain a more in-depth understanding of what influences levels of research activity. Focus groups were used to facilitate open and honest discussion without the pressure to answer every question. Individual interviews were used to ensure that we could collect the perspectives of senior leaders and decision makers who were generally unable to participate in the group sessions. We commissioned a rapid evidence review of potential models for a local government research system (full review report is available at https://actearly.org.uk/wp-content/uploads/2020/12/Rapid-review-report.pdf). Subsequently, we developed a typology of local authority research activity ( Figure 1) which was reviewed by our interview participants, and more widely by other local authorities and networks in our region (Yorkshire and Humber). We completed scoping reviews of use of evidence in decision making and training opportunities within BMDC, as well as existing infrastructure support for local government research.

### Data collection and analyses

a) BMDC online staff survey

Between 17 September and 9 October 2020, an online google survey was sent internally to a convenience sample of 600 randomly selected employees across all levels within BMDC (facilitated by RS). Inclusion was based on employment and email access with BMDC with no other exclusion criteria. Random sampling and repeat reminders were issued, and we reviewed responses targeting groups with low response to ensure a representative sample and to minimise bias. We assessed knowledge of sources of evidence, use of research, research commissioning and current or past research funding received by BMDC. A total of 197 employees from a range of BMDC departments completed the survey and results were presented as proportions of the sample responding using Microsoft Excel 2010.

b) Focus groups

In total, two 1-hour focus groups were held via Zoom video conferencing during September 2020 with a total of 11 participants working at senior levels across a range of departments within BMDC (e.g. BMDC leadership, associate directors, elected members, public health senior leadership team). Participants were selected using a convenience sample approach and invited by internal email (facilitated by RS). Group interviews were undertaken and guided by KC (Research Fellow) and were video recorded. We explored participants’ understanding of research, the barriers and enablers to them using research, and discussed what would be needed to sustain a local research system. Data were analysed by KC and SB using Thematic Analysis ^8^.

c) Individual interviews

We interviewed via Zoom video conferencing, 11 further local authority staff and members including key directorate leaders, elected members, the Council Leader and the Chief Executive during August and September 2020. A convenience sampling approach was used to recruit participants working at senior levels across a range of BMDC departments (e.g. BMDC leadership, associate directors, elected members, public health senior leadership team). Individual 1-hour interviews were undertaken and guided by KC (Research Fellow). All interviews were video recorded. We explored understanding of research and evidence, barriers and enablers to how research is used within BMDC and gathered views on developing and sustaining research activity. Data were analysed by KC and SB using Thematic Analysis ^8^.

d) Rapid literature review of existing models

A rapid review of existing published models of local authority-based research systems was commissioned from the School of Health and Related Research (ScHARR), University of Sheffield (full review available in the *Extended data* ^9^).

e) Pilot use of our local authority research activity typology

During the interview sessions, we asked focus group and individual interview participants to benchmark BMDC’s research activity using our typology tool ( Figure 1). We also shared the tool and sought comments from two other local authorities in our region (Doncaster and Wakefield) and the regional PaRC (PHE led regional public health research hub). We report the most common level and range.

f) Documentary review of decision making

We explored the use of research in BMDC decision making. Two researchers (Jwes, LL) independently reviewed minutes of all meetings for two of the council’s senior strategic boards – the Bradford and Airedale Health and Wellbeing Board (HWB) and the Integration and Change Board (ICB) held between 1 ^st^ January 2019 and 31 ^st^ March 2020, to identify minuted examples of the use of research in any discussions or decision making. Both researchers checked their findings to confirm agreement.

g) Information regarding research staff, research training and skills development, and career development within BMDC was provided via email (due to home-working and COVID-19 restrictions) as a narrative by the BMDC Director of Public Health (Co-applicant) and BMDC Director of Policy and Performance (Co-applicant).

h) We collected information around support for local authority research from the Research Design Service (RDS) Yorkshire and Humber and the national NIHR Centre for Engagement and Dissemination (CED) between September and November 2020 and report a narrative summary.

### Consent

Consent for qualitative interviews was taken verbally and video recorded at the start of the interview in line with the ethics approval for this project. Implied consent for the online survey was assumed on completion and submission of the questionnaire.

### Ethics

This project was approved by the University of York Health Sciences Research Governance Committee.

## Results

Below we describe the results from the varied components of this project. First, we report the findings of our quantitative and qualitative research (survey and interviews), then a summary of the testing of our proposed typology followed by a descriptive summary of the use of research in decision making, a summary of our rapid review of existing models, a descriptive summary of our scoping of capacity and lastly, a summary of external research infrastructure available to local authorities in England.

### a) Quantitative staff survey and qualitative focus group and individual interviews

The key findings from our quantitative survey and qualitative interviews (focus groups and individual) are described below ^9^. First, we report views on current research activity, and second, we summarise the findings within four main themes that emerged from the data: leadership, resource and capacity, culture and partnerships using unidentified quotes from participants.

***Current research landscape.*** Generally, participants felt that research and evidence was used and valued across BMDC. Research was described as “ *a really broad church”* which included BMDC commissioned research and research where the council collaborated with partners. Most participants stated that using research and evidence is expected and is part of what they do. In the staff survey, 73% of respondents strongly agreed or agreed that using research evidence was part of their role and of these, 82% reported using research evidence (including in house research) to help inform or develop policies, projects, interventions or services. Participants were not aware of a clear plan or policy for how research is used within BMDC; one participant noted:

*“We don’t have a programme of work around research and we don’t have a nominated research lead and we don’t have kind of tick lists of research and we don’t have anybody pursuing research opportunities outside of their core work. So … it could be more, higher profile and more coordinated and also expanded out to the broader Council”.*

Some felt that research was academic and complicated and spoke of the need to simplify and ‘ *demystify research’* with simpler messaging and communication including definitions, language, training, processes, and messaging around benefits of using research:

“ *[research] needs to be more approachable. I think research is a scary word for people”.*

*“Research is viewed as academic - some of the boundaries around using and applying research need to be broken down. The benefits of primary/secondary research undertaken by the BMDC need to be made more obvious”.*

There was a lack of knowledge about how to find relevant and current evidence and participants wanted this to be easier. Barriers to using evidence, such as being unable to access peer review journals through BMDC IT systems, were also identified; only 31% of online survey respondents used peer reviewed journal papers and just 12% reported being able to access them online at BMDC. 

Internal data sharing processes were described as a barrier to research and participants noted that there were no mechanisms in place to allow sharing of research and evidence across departments. This sometimes led to duplication and silo working:

" *I have found it difficult to identify which person/department has access to the information and research that may be useful, and trying to form any lasting relationships between departments has in my experience been unsuccessful. Knowing who to ask for things has been a huge barrier for me. I think sometimes members of staff are unwilling to share their work and what they know, but this relates more to internal pieces of research and studies of information”.*

***Leadership.*** Leadership was considered crucial to a research system. Participants recognised the need to get ‘buy in’ across the organisation:

“ *When staff are very, very busy they do struggle to give up their time to get involved in something like that [research]. So it needs some leadership and gentle persuasion to sit behind it*”.

It was also noted that buy in at a political leadership level was required to commit to the principle of being evidence-led. Having this clear commitment to research both at a management and political level, was considered an important part of any BMDC research system.

“ *Some kind of overall policy sign-off from our politicians that that’s the strategic direction they wanted us to follow and that they understood that that meant that our staff and even some of our resources will be out in that direction”.*

A policy or system for using research was considered helpful but should be appropriate and achievable rather than bureaucratic. There were several comments that this should be outcomes based – indicating what works and how to intervene rather than just describing the problem.

***Resource and capacity.*** Capacity including time, skills and training and money was frequently highlighted. Many participants felt they did not have the time to engage in research, especially as research is not generally prioritised. At a more strategic level, no time was given to planning future research needs which was seen as “firefighting” and a reliance on doing things as they have been done previously:

*“You tend to buy what you’ve always bought because the council hasn’t got capacity to think, well, what do you think we should be buying, or what research should we be doing to find out how we should organise these…services next time the contract comes up”.*

“ *In the past we used research to steer our work, now all we seem to do is be reactive to situation. I feel this is due to job cuts as people are just getting on with things every day and no time to research or reflect”*.

Skills and training were reported as variable across the organisation, but there was agreement that a range of research skills would be needed if BMDC was to increase its use of research and that basic research literacy is lacking in many departments. There was a consistent message around the challenge of how to prioritise funding, or generate funding to support research capacity but recognition that good research could lead to cost savings and so could be cost effective.

*“We could prioritise what we want to deal with, which I think the politicians and the top of our organisation find very, very difficult to do. Or we just have to kind of keep spinning plates, or we invest in it more but we just do not have the resources to invest in, in it, we just don’t and, and I think it’s going to get tighter more than, more than… because of COVID and because of the pressures that come through COVID”.*

***Research culture.*** BMDC was not considered homogenous in terms of its research use, attitudes or literacy. The council was described as “ *lots of different types of organisations in one”,* and as having *“lots of subcultures”*. Varying levels of engagement and readiness for research were reported across departments:

*“I work in public health - so clearly evidence is important! It’s not something which is appreciated or recognised across other departments. It’s not within their culture/approach to work. So there’s something about raising awareness, increasing skills and capacity, and showcasing how important and how it can make a difference”.*

BMDC was described by some as being risk averse, in terms of the scale of interventions implemented and around data sharing activities, both internally and with third parties. Despite this there was a clear ambition at senior levels for research to be core to BMDC’s work:

 “ *The level of ambition is high but the level of resource to deliver against that ambition is low*”.

This contradiction was recognised by senior figures, who acknowledged that, whilst not perfect, the use of research and evidence in BMDC was improving and was empowering decision making:

“ *Given the evidence, it’s easier to make more difficult political decisions and I think sometimes politicians don’t have all of the evidence to make those difficult decisions*”.

***Partnerships.*** Partnership working with universities and other research organisations was common, with lots of examples highlighted by participants and there was enthusiasm to build on existing partnerships and increase activity and opportunities for BMDC to contribute more fully: 

“ *… we’ve got a great asset ..in the Institute of Health Research that you’re sitting in, and Born in Bradford and we’re very lucky in Bradford in terms of having that, and we do use that but not, not as much as we could do to match our kind of overall ambitions, just because of both the time and the, the resource…*”.

Voluntary, community and social enterprises (VCSE) were noted as offering important partnerships, not just in terms of service delivery but also as research and evaluation partners. Of those online survey respondents who stated they had been involved in commissioning research, 52% (n=17) used a research organisation, 36% (n=12) commissioned a university, and 24% (n=8) commissioned a local VCSE organisation.

### b) Testing of our draft local authority research activity typology

We asked focus group and individual interview participants to rank BMDC using the typology ( Figure 1). The most commonly reported level was 2 (range 1–3). External colleagues found it straightforward and a useful indicator of research activity, but commented levels may be estimated differently across internal directorates where some may be more research active than others.

### c) Scoping of research use in BMDC decision making

We reviewed minutes of all meetings for two of the council’s senior strategic boards – the Bradford and Airedale Health and Wellbeing Board (HWB) and the senior management level Integration and Change Board (ICB), held between 1 ^st^ January 2019 and 31 ^st^ March 2020. HWB minutes included multiple references to evaluation of local projects, though no formal record of using evidence in decision making. There was a standing ICB agenda item on research, and throughout the ICB minutes we identified statements underlining the priority of strengthening the application of research in practice for example how research was a “catalyst for change” and “more research activity and evidence means better staff recruitment and better outcomes”. No research references aligned to specific decision making were identified.

### d) Rapid evidence review of existing models

Our rapid review of existing published models of local authority-based research systems was undertaken by ScHARR, University of Sheffield and found nine distinct model types of which four were UK based. Briefly, the overall quality of evaluation of models was low. They varied in how they considered development of research capacity and capabilities within local government and had different approaches to facilitating the choosing (finding and accessing), using (to inform decision making) and producing of research (related to local government decisions, activities and needs). Models shared similar components, most commonly leadership and research culture, but were based on different assumptions around power and governance structures, degree of location/co-location, physical presence and ownership of each system, and the respective roles of academia and local government. The most recent and most substantive UK model was the Local Authority Champions of Research (LACoR) Logic Model ^10^ which fits well with the four themes that emerged from our fieldwork (leadership, resource and capacity, culture and partnerships). It is underpinned by a systems thinking approach which aligns with a range of research programmes in Bradford which are based on complexity thinking, including the NIHR PHR funded evaluation of the health impact of a city-wide system approach to improve air quality and the UK Prevention Research Partnership (UKPRP) ActEarly Consortium’s whole system model of prevention ^11^.

### e) Scoping of local government research capacity and career development

Two members of our project team were working within BMDC and reported that there was no specific BMDC research staff and where staff had research training or knowledge, they lacked the time to use it. The NIHR CRN Yorkshire and Humber has funded a BMDC-based data analyst for 12 months to help develop linked datasets for the ActEarly consortium and this was seen as having driven progress in data linkage and editing of education and health datasets for use by researchers. More generally, it was suggested that improving knowledge around basic research principles, ethics and governance (i.e. safe handling of data) would engender a more research friendly environment, and introducing critical appraisal skills would be useful for policy development, so that staff could better choose and use evidence.

### f) Scoping of existing research infrastructure support for local government research activity

*NIHR Clinical Research Network (CRN):* The NIHR CRN’s remit was widened in 2018 to include public health and social care studies, but its activity and performance management was found to still be clinically focused with many public health researchers having limited knowledge and understanding of the network. The LCRN funded data analyst post at BMDC was an example of how the network can make progress towards developing support for public health and other non-recruiting studies. 

*NIHR Applied Research Collaboration (ARC) Yorkshire and Humber:* The NIHR has asked all ARCs to ensure that public health, mental health and social care are embedded across the work programme and that key stakeholders from these areas are involved to ensure impact in these areas. ARC Yorkshire and Humber reported actively engaging local authorities in collaborative research projects, and facilitates research relationships between local government and academia. Three Local Authorities (Doncaster, Leeds and Bradford) are current ARC member organisations. 

*NIHR Research Design Service (RDS) Yorkshire and Humber:* There was no specific strategy for local authorities but supporting more public health research was reported as one of the national RDS priorities and the service is further developing the support offered to local authority colleagues by working with the pilot NIHR Public Health Research Applications and Design Assistance (PHRADA) service and a RDS Partnership Group.

*NIHR Centre for Engagement and Dissemination (CED):* The CED reported linking with the Public Health England (PHE) librarian network as a way of providing updates for public health staff in local government.

The *NIHR Academy:* The Academy provided information on two new schemes aimed at local authority staff due to launch in early 2021 which will support a combined practitioner/researcher role at pre-doctoral and doctoral level in Local Authorities.

## Discussion and development of a local research system

### Summary and discussion of key findings from data collection and reviews

We found that BMDC demonstrates features which broadly correspond to level 2 in our typology ( Figure 1). It is responsive and supportive when approached by academic partners, but less likely to create and use research independently. The importance of research is mostly well recognised with some senior support, but there are challenges to research activity around resources, politics, understanding and skills. External support from NIHR infrastructure is slowly adapting to the local government environment but much more work is needed to shift the centre of gravity towards public health, local government and the community more generally. We used a random sample for our online staff survey, this may have meant that only those staff working in roles with frequent use of email communications and with online access were able to complete the survey. However, we anticipated that these staff would be those most likely to be engaged in research which was the focus of our survey questions. Often research language and what constitutes evidence outside health and care environments is different to that within them, and it is possible that what local government staff categorise as research or evidence may be different to what is understood to be research in a health environment. We piloted our questions (survey and interview) prior to undertaking the study with our BMDC co-applicants (PW and RS) to minimise misunderstanding. We were also interested to identify these differences in language and understanding as part of this project. Some areas of our data collection were impacted by the COVID-19 pandemic and related restrictions, for example all interviews were undertaken at a time when participants were working in a home environment using video conferencing, which required a stable internet connection and may have excluded those unable to provide that. However, our targeted approaches to interview participants and repeated reminders to those under-represented aimed to minimise risk of bias and resulted in this having a minimal effect on our range of participants. Only publicly online available information was examined for the review of BMDC decision making which could potentially have provided a limited view of research use in BMDC decision making, however our aim was to scope research activity rather than audit all activity, with the aim of informing a research system proposal and we used a broad range of methods to do that (i.e. survey, interviews, review of activity, review of NIHR support, rapid literature review).

### An adapted research system model for local government

**Figure 2.**
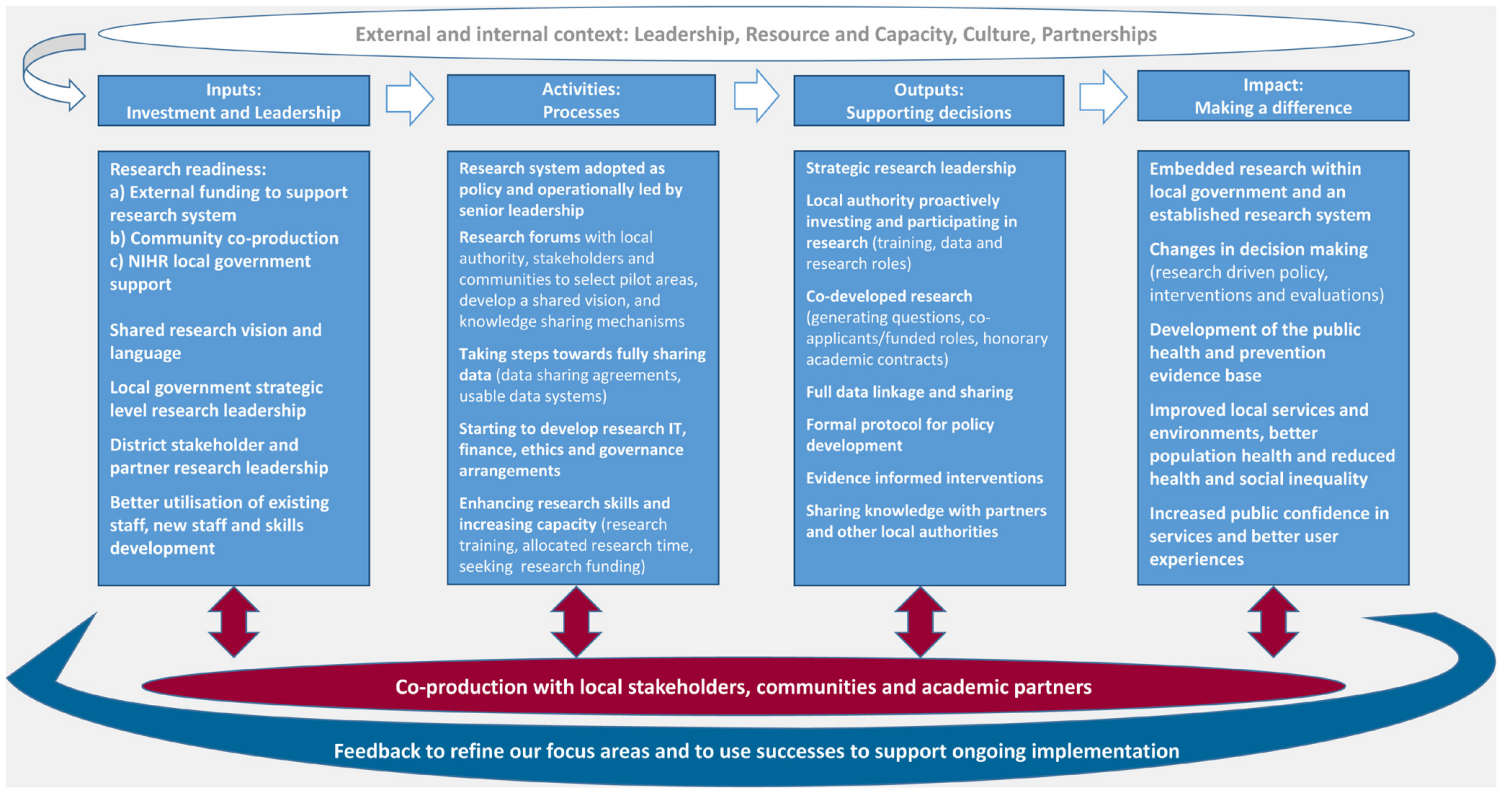
Proposed local research system model (adapted from the Local Authority Champions of Research (LACoR) logic model). NIHR, National Institute for Health Research.

The LACoR Logic Model ^10^ was the best fit for our context, however, the scale, depth of application, embeddedness and independence is at a very early stage in Bradford (features that the model does not include). For example, BMDC has contributed to data sharing agreements, collaborations and co-production when approached by others but is some way from leading these activities. We propose a local research system based on the LACoR model but that recognises the depth and independence of inputs and outputs, as well as the research activity and networks already established in many local authorities. Our adapted model ( Figure 2) aligns with the priority themes that emerged from our survey and interviews (leadership, resource and capacity, culture and partnerships), incorporates the components of our typology, and is a model for the local system rather than specifically the local authority. It is deliberately concise, as through our fieldwork, we found that people would like to see simple messaging and processes for research.

### Delivering a local research system

1) ***System research readiness***

In this scoping project, we identified a number of conditions important to ensuring readiness for a local research system:

*A shared vision, language and understanding of research:* We found varied accounts of what is accepted as evidence or research. Local government is a political environment subject to political cycles and leadership changes. Elected members respond to their communities which means that research evidence is only one form of evidence used to make decisions, and views on its importance and value can be mixed. Similarly, different understanding of what is ‘research evidence’ exists not just between local authorities and partners, but also within them. We suggest the need for a shared research vision, understanding and language with local government, academic and local partners, infrastructure and funding representatives, and local communities so that the wide-ranging disciplinary areas within local government can connect internally and externally to become more research active.

*Additional external resource*: Government funding for local authorities fell by almost 50% in real terms between 2011 and 2018 ^12^ and the COVID-19 pandemic has brought further challenges. For local authorities to move from being a responsive research partner to a more proactive research organisation, significant resource is needed to support and sustain a research system in local government. External investment in a research skilled workforce (collaborating with and supported by existing infrastructure and academic partners), research and development infrastructure (data systems, IT research related software, access to online research, research finance support), governance and ethics arrangements, and co-production activity is needed so that local authorities can choose and use research, and fully participate in generating and delivering research alongside academic partners.

*Co-production with stakeholders and communities:* In Bradford, there are well-established community assets on which co-produced research with stakeholders and communities could be developed, for example by embedding citizen science approaches and expanding our existing community research advisory groups within existing local authority structures, networks and activities across the local system. This may be less well-developed in other local authority areas and we include community co-production in our model as a formal component of a research system. 

*Existing research infrastructure:* For the development and sustainability of local authority research systems, the existing infrastructure provided by the NIHR will need some rebalancing of clinical research support with the complex non-clinical environment of local government. *NIHR CRN* support could be improved by increasing the network’s resource allocation to local government, and by developing new mechanisms of support that work for non-clinical and non-recruiting research, for example support for data access, linkage and sharing. *NIHR ARCs* should be encouraged to include local government in their steering groups and address local authority health–related priorities. *NIHR RDS* could further expand its public health expertise by a wider NIHR requirement for NIHR Public Health Research Programme principle investigators and NIHR Senior Investigators working in public health, to provide expert support to those seeking local government and public health support from the RDS. *NIHR CED* has an opportunity to drive knowledge mobilisation and exchange between local authorities to support development of and access to the public health evidence base, for example, by providing evidence summaries for the Local Government Association, Association of Directors of Public Health, Local Authorities Research and Intelligence Association (LARIA). The development of a registry of local authority research (similar to the NIHR Be Part of Research register) could be considered. *NIHR Academy* has launched two new fellowship programmes for local government staff in 2021 and is developing secondment opportunities for academics to work within local government ( https://www.nihr.ac.uk/explore-nihr/funding-programmes/nihr-local-authority-academic-fellowship-programme-and-associated-opportunities.htm). In addition to adapting existing infrastructure, in autumn 2021 the NIHR is launching new infrastructure funding for local government through Health Determinants Research Collaborations (HDRCs) which will support the development of infrastructure to help local authorities become more research-active (call launch September 2021).

***2) Implementing a local research system***

Our system research readiness conditions describe what needs to change, and below we outline the actions required of local authorities to start to implement our proposed model:

1. Commitment for a local research system should be sought from senior local authority leadership and other leaders across the local system. The development of a research system will need to be adopted as policy by the council, be accountable to the council at executive level, and operationally led by senior council Executive members. Research utilisation and evaluation will become a core part of local government leadership development, including how to manage any staff that may resist efforts to evaluate a project or enable data sharing. Locally, research ecosystems are complex and difficult to navigate, therefore as well as being adopted internally within the council, the system also needs to engage and capture the wider local system so that all organisations and partners that have the potential to influence wider determinants of health, can play their part in choosing, using an degenerating research evidence. 

2. A pilot of our adapted model is suggested using two areas of high priority for the local authority in the first instance. This can demonstrate the power of connected local datasets that link system wide factors relevant to a range of local authority departments and partner organisations. It will also demonstrate which interventions work, impacts, and potential budget savings that can be fedback across the local authority and the local system to generate interest for roll-out of the model more widely. Consultation with leaders and communities through research forums will facilitate consensus and allow the selection of pilot topic areas which are important to public health and are impacted by system wide factors under the control of a range of local authority departments (e.g. transport, education, environment), and can encourage wide engagement across the council.

3. The activities identified in our adapted model ( Figure 2) should be prioritised, for example starting to develop full data sharing, enhancing research skills and increasing capacity through new staff and allocated research time for existing staff, supported by academic partnership and support from the existing NIHR infrastructure.

4. The application of adapted improvement methodology to iteratively implement the action plan. Applying this approach will acknowledge that the organisation contains disciplines at different stages on the 'evidence-based practice' journey and that tailored approaches will be needed. As development of the research system progresses, areas that need to change to move up the typology will continue to be identified and will provide learning for progression to the next level. 

5. Formal evaluation of progress against the outputs and outcomes in our adapted model should be embedded from the outset, for example changes in decision making and evidence informed policy making. Evaluation should also include the process of embedding research in the local authority for example, over time the research system leadership, resource and capacity, culture and partnerships will evolve and be refined. A “research on research” study within the research system would enable a better understanding of this process and its influence on the local system.

## Conclusion

In this scoping project we have identified both the challenges to, and the strong appetite for a local research system. Using our findings, we have developed a generalisable model for a local authority research system that can underpin a whole system local government research framework providing infrastructure and an evidence culture to support the development and expansion of sustainable local government research activity.

## Data availability

### Underlying data

Our online survey and full rapid review are available via the Harvard Dataverse (below). This link also includes limited interview information. Full transcripts have not been made publicly available to protect the identity of those taking part. However, we are happy to accept requests for detailed transcripts where we can be assured that participant anonymity can be protected. Please forward requests to actearly@bthft.nhs.uk.

Harvard Dataverse: A model for local government health determinants research: Underlying data & methods (survey; interviews). https://doi.org/10.7910/DVN/BCYXZZ ^9^.

This project includes the following underlying data:

- WOR LARS online staff survey (Responses).xlsx

### Extended data

Harvard Dataverse: A model for local government health determinants research: Underlying data & methods (survey; interviews). https://doi.org/10.7910/DVN/BCYXZZ ^9^.

This project includes the following extended data:

- WOR Model for local government health determinats research~underlying data updated 13Sept.pdf (quantitative online staff survey; qualitative focus group and individual interview schedules; rapid review of existing models)

Data are available under the terms of the Creative Commons Zero "No rights reserved" data waiver (CC0 1.0 Public domain dedication).
