# Supplementary material for: Developing a model for health determinants research within local government: lessons from a large, urban local authority
Source: Wellcome Open Res. 2022 Jun 21;6:276. Originally published 2021 Oct 15. [Version 2] doi: 10.12688/wellcomeopenres.17195.2 (PMC9277198; doi:10.12688/wellcomeopenres.17195.2)
Supplement: Peer review history

# Peer review: Reviewer response for version 2

**DOI:** 10.21956/wellcomeopenres.19921.r51172

**Published:** 2022-07-12

**Authors:** Naia Hernantes

**Affiliations:** 1 Faculty of Medicine and Nursing, Department of Nursing, University of the Basque Country (UPV/EHU), Donostia-San Sebastian, Spain

Many thanks to the authors for their efforts in making the changes and the answers provided.

 All the specified details help to a better understanding of the manuscript.

Is the work clearly and accurately presented and does it cite the current literature?

Partly

If applicable, is the statistical analysis and its interpretation appropriate?

I cannot comment. A qualified statistician is required.

Are all the source data underlying the results available to ensure full reproducibility?

Yes

Is the study design appropriate and is the work technically sound?

Partly

Are the conclusions drawn adequately supported by the results?

Partly

Are sufficient details of methods and analysis provided to allow replication by others?

No

Reviewer Expertise:

Capacity building for Intersectoral Health Promotion at local governments, mixed methods.

I confirm that I have read this submission and believe that I have an appropriate level of expertise to confirm that it is of an acceptable scientific standard.

# Peer review: Reviewer response for version 1

**DOI:** 10.21956/wellcomeopenres.18999.r49296

**Published:** 2022-04-25

**Authors:** Yitka Graham

**Affiliations:** 1 Faculty of Health Sciences and Wellbeing, University of Sunderland, Sunderland, UK

This is a thorough study which clearly addresses a current issue issue within local government and research.

**Introduction/background**

 This is well written, with the research question and rationale for the research clearly articulated.

**Methods**

 These were appropriate to answer the research question, the methodology is again articulated and justified well.

 Figure 1 provides a useful and pragmatic typology

 Consent and ethical approval processes are stated.

**Results**

 The results are well presented, but the section could do with an introductory sentence to introduce the six sections to guide the reader

 a) Results are presented with examples of in-vivo quotations to support views of participants and the constructed themes from the data analysis.

 b) reads well, no comments

 c) reads well, no comments

 d) well justified and explained

 e) reads well. no comments

 f) reads well, no comments

**Discussion**

 The discussion is detailed and divided into appropriate headings so this section flows well. Strengths and limitations are discussed, as are recommendations.

Is the work clearly and accurately presented and does it cite the current literature?

Yes

If applicable, is the statistical analysis and its interpretation appropriate?

Not applicable

Are all the source data underlying the results available to ensure full reproducibility?

Yes

Is the study design appropriate and is the work technically sound?

Yes

Are the conclusions drawn adequately supported by the results?

Yes

Are sufficient details of methods and analysis provided to allow replication by others?

Yes

Reviewer Expertise:

Qualitative methodologies, applied health research

I confirm that I have read this submission and believe that I have an appropriate level of expertise to confirm that it is of an acceptable scientific standard.

# Response

**Published:** 2022-06-09

**Authors:** Jane West

**Affiliations:** Born in Bradford, UK

We thank the reviewer for taking the time to review our article and for the useful comments. We have added an introductory sentence to the Results section to guide the reader as suggested.

# Peer review: Reviewer response for version 1

**DOI:** 10.21956/wellcomeopenres.18999.r47278

**Published:** 2021-12-20

**Authors:** Naia Hernantes

**Affiliations:** 1 Faculty of Medicine and Nursing, Department of Nursing, University of the Basque Country (UPV/EHU), Donostia-San Sebastian, Spain

The research field addressed in this study is very relevant and challenging at the same time. Overall, the results obtained in this study are very enriching for the health promotion field at local governments.

 However, the authors should address some aspects:

- It would be convenient to structure the abstract section in a coherent way with the article; in the methods section, the methods themselves are mixed with the objectives of the study. It would be helpful to identify them separately.
- Relating to the methods section: On the one hand, has the use of all of the methods been framed under a specific methodology? On the other hand, focus groups and group interviews are used interchangeably, but they are methods that pursue very different objectives. Finally, could we have a brief description of the methods used in the rapid review? And how were quantitative and qualitative data integrated?
- I recommend restructuring the entire results section based on the objectives of the study. Currently, results are structured based on the methods used, but those methods only are the way to achieve the objectives of the study.
- It would be interesting to have a table of participants, with sociodemographic data, who have participated in each of the methods used.
- Figure 2 appears in the discussion, however, according to the objectives of the study, it should be included in the results section, since this figure is a result in itself.
- I would suggest moving the discussion part titled "Delivering a local research system 2) Implementing a local research system" to the conclusions as future recommendations.

Is the work clearly and accurately presented and does it cite the current literature?

Partly

If applicable, is the statistical analysis and its interpretation appropriate?

I cannot comment. A qualified statistician is required.

Are all the source data underlying the results available to ensure full reproducibility?

Yes

Is the study design appropriate and is the work technically sound?

Partly

Are the conclusions drawn adequately supported by the results?

Partly

Are sufficient details of methods and analysis provided to allow replication by others?

No

Reviewer Expertise:

Capacity building for Intersectoral Health Promotion at local governments, mixed methods.

I confirm that I have read this submission and believe that I have an appropriate level of expertise to confirm that it is of an acceptable scientific standard, however I have significant reservations, as outlined above.

# Response

**Published:** 2022-06-09

**Authors:** Jane West

**Affiliations:** Born in Bradford, UK

We thank the reviewer for taking the time to undertake the review and for their useful comments. Even though this article is listed as a Research Article which was an editorial team decision, this piece of work was a multi-component scoping project with some research components (online survey, qualitative interviews, rapid evidence review). The other elements of the project were scoping and descriptive exercises rather than research and we apologise if this has led to some confusion.

1. The abstract currently summarises the non-research and research components of this project, as such we feel that the abstract currently captures all these different elements, however, we have clarified that in the text.
2. We understand that the methods section also incorporates the varied parts of this project and we have added to the start of this section to help clarify this. We have now added a link to the full details of the rapid review undertaken by the University of Sheffield. We have also clarified the justification for the qualitative research methods used.
3. The Results section is currently structured to align with the Methods section. Again, a number of components of this study were not ‘research’, rather they were exploratory scoping exercises to capture current practices in local government.
4. We have added a sentence to the start of the Results section (as requested by Reviewer 2) to clarify the order and content of this section.
5. Only two areas of this work involved participants (the online survey and the qualitative interviews) and these were all local authority staff members. Only very limited identifying information was collected meaning that sociodemographic data was not collected. For the online survey the project team did not have access to the job role and name of any responders to the survey (only RS internal to the local authority had this access in line with ethics approval). For the qualitative interviews, only the interviewer (KC) and RS had access to names, contact emails and job roles to ensure that responses were treated in confidence (again to comply with ethics approval). As such, it was not possible to provide any sociodemographic information.
6. Our proposed model (Figure 2) was developed following the rapid review and we feel that this is best placed in the discussion section however, we are happy to take editorial advice and move to the results section if required.
7. As research activity remains limited in most local authorities in England, we were anxious not to suggest that our conclusions should be seen as something that local authorities should be doing (i.e. “recommendations”). We hope the report is a reference for local authorities that are exploring the potential to develop their research activity in the future. However, again we are happy to follow the advice of the editorial team on this point.
